# Running on Empty: Of Hypopinealism and Human Seasonality

**DOI:** 10.3389/fphar.2021.681582

**Published:** 2021-10-15

**Authors:** Dieter Kunz, Amely Wahnschaffe, Nina Kaempfe, Richard Mahlberg

**Affiliations:** ^1^ Clinic for Sleep and Chronomedicine, St. Hedwig Hospital Berlin, Berlin, Germany; ^2^ Institute of Physiology, Charité – Universitätsmedizin Berlin, Berlin, Germany

**Keywords:** pineal calcification, melatonin, hypopinealism, seasonality, SAD, seasonal affective disorder

## Abstract

The pineal hormone melatonin is the natural transducer of the environmental light–dark signal to the body. Although the responsiveness to photoperiod is well-conserved in humans, only about 25 percent of the human population experiences seasonal changes in behavior. As a consequence, humans seem to have adapted—at least partly—to the seasonal changes in day length. The aim of the study was to demonstrate that the individual melatonin deficit marker DOC (degree of pineal calcification) is related to variation of seasonal phenomena in humans. Out of 3,011 patients in which cranial computer tomography (cCT) was performed for diagnostic reasons, 97 consecutive “healthy” subjects (43 female, 54 male; age 18–68 yrs, mean ± SD: 35.0 ± 13.1) were included. Exclusion criteria were pathological finding in cCT, acute/chronic illness including alcohol/drug abuse, shift work, and medication, which are known to influence melatonin excretion. The degree of pineal calcification (DOC) was semiquantitatively determined using the previously validated method. The Seasonal Pattern Assessment Questionnaire (SPAQ) was performed in a telephone interview. Twenty-six subjects fulfilled the criteria for seasonal affective disorder (SAD) or subsyndromal (S) SAD. Seasonality was more pronounced in women than in men (SPAQ seasonality score: 7.8 ± 4.0 vs. 4.9 ± 4.5; *p* = 0.001) and negatively and significantly associated with age (r = −0.178; *p* = 0.04). The subjective sleep length significantly varied between seasons (one-way repeated measures ANOVA: F = 45.75; *p* < 0.0001), with sleep during winter being 53 min (±70 min) longer than during summer. Controlling for age, the total seasonality score was negatively and significantly associated with DOC (r_94_ = −0.214; *p* = 0.036). Data confirm earlier studies with respect to distribution of seasonality with sex and age. The survival of seasonality in the sleep length of people living in an urban environment underlines functionality of the circadian timing system in modern societies. Moreover, data confirm for the first time that diminished experience of seasonality in behavior is associated with a reduced individual capacity to produce melatonin.

## Introduction

“The circadian clock is involved in every piece of human physiology; it covers everything from emotions to endocrinology to metabolism.” In 2017, the Nobel Prize committee awarded discoveries in circadian rhythms and therewith acknowledged the insight on the ubiquity of circadian rhythms in life on earth including humans (Burki, 2017). In humans, at least 50 percent of all molecular mechanisms have been proven to exert their own circadian rhythm (Clayton et al., 2002; Allada and Bass, 2021). Synchronization of internal circadian rhythms with local time of the outside 24-h world is achieved by zeitgebers (Pittendrigh, 1993), in humans primarily light and dark (Berson, 2003).

Except close to the equator, the length of the light–dark signal varies over the 24-h day. The natural transducer of the light–dark signal to the body is the pineal hormone melatonin (Bartness and Goldman, 1989). In animals, changes in the duration of melatonin excretion over the year trigger seasonal phenomena such as breeding, migration, and hibernation (Pittendrigh, 1993).

In humans, photoperiod responsiveness is well-conserved but seems to be more complex (Wehr et al., 1993): in experimental settings (1 week of 14-h vs. 1 week of 8-h bright light per day), melatonin excretion times over the 24-h day covary with the winter–summer photoperiod to the magnitude of 6 h (Wehr, 1997); patients suffering from seasonal affective disorder show larger summer–winter variation in melatonin excretion rates then subjects without such seasonality (Wehr et al., 2001); on the other hand, in an urban environment, only a part of women show seasonal changes in melatonin excretion rates, whereas most men do not (Wehr, 1997); seasonality of behavior seems to be preserved in young adults and partly in older women, but not in older men (Kasper et al., 1989; Magnusson and Boivin, 2003); moreover, in large epidemiologic studies, only about 25 percent of the human population experience seasonal changes in behavior to an extent causing at least mild problems (Kasper et al., 1989; Magnusson and Boivin, 2003). It seems as if humans have either adapted—at least partly—to the seasonal changes in day length or the introduction of artificial light as the major light source may hinder the effects of natural light to induce seasonal changes. The mechanisms behind are not understood.

We have earlier introduced the DOC as an individual melatonin deficit marker in humans (Kunz et al., 1999). First, results suggested that increasing DOC scores are related to experiencing fewer seasonal phenomena in humans (Kunz et al., 2001). The aim of the study was to show that in an urban environment, individually low melatonin, as indicated by high DOC scores, is associated with low seasonal variation in human behavior.

## Materials and Methods

### Sample Selection

In order to collect data from a “healthy population,” hospital files of 3,011 consecutive patients were checked. Patients had attended the emergency room of Charité – Universitätsmedizin Berlin, Campus Mitte, from January 01, 2003 to June 08, 2006, for cranial bruises or unspecific neurological symptoms such as dizziness, swindle, headache, or fever and had received a cranial computed tomography scan (cCT) for diagnostic reasons.

The inclusion criterium was age between 18 and 80 years. The exclusion criterium was performed in several steps. **Step 1**: After inspection of cCT reports, a total of 1947 patients were excluded because of age (*n* = 170); acute/chronic illnesses including neurologic illness (*n* = 1,185), psychiatric illness, and alcohol abuse (*n* = 148), extracranial tumors (*n* = 164); cardiac diseases (*n* = 68); diabetes mellitus (*n* = 16); HIV (*n* = 22); reanimation (*n* = 21); illicit drug intoxication (*n* = 4); foreign tourists (*n* = 7); cCT not adequate (*n* = 60); and further reasons (e.g., betablockers, benzodiazepines, antidepressants, and antiphlogistics; *n* = 82). After step 1, 1,064 patients were left. **Step 2:** After inspection of hospital files (MedVision), a total of 589 further patients were excluded because of neurologic diseases (*n* = 68), psychiatric diseases (*n* = 31), extracranial tumor (*n* = 20), cardiac diseases (*n* = 21), diabetes mellitus (*n* = 12), HIV (*n* = 2), reanimation (*n* = 1), illicit drug intoxication (*n* = 3), foreign tourists (*n* = 3), dead (*n* = 1), medication (*n* = 2), pineal gland area in cCT not in 2 mm slices (*n* = 154), no record in MedVision files (*n* = 22), no phone number in files (*n* = 210), or further reasons (*n* = 39). **Step 3:** The remaining 475 patients were phoned, and another 365 patients were excluded because they could not be reached after at least two attempts (*n* = 166), refusal to participate (*n* = 19), lack of language skills (*n* = 24), and additional information from telephone anamnesis: medication (*n* = 56), neurologic (*n* = 42) or psychiatric illnesses (*n* = 7), alcohol abuse (*n* = 12), ongoing or at least until 2 years ago shift work (*n* = 16), or further reasons (*n* = 23). **Step 4:** Of the remaining 110 patients, cCT images were checked and 13 patients were excluded because cCT scans were determined not judgeable, for example, because of movement artifact or the pineal gland could not clearly be distinguished from arteria pinealis. Finally, 97 patients were included in this study.

### Seasonal Pattern Assessment Questionnaire

In a telephone interview, patients were personally interviewed during the period of February 01, 2006 until June 30, 2006. The Seasonal Pattern Assessment Questionnaire (SPAQ) (Rosenthal and Wehr, 1984) was used. For analysis, three variables from the SPAQ were calculated as follows:

1. The total SPAQ seasonality score (0–24) is a sum score derived from the intensity of seasonal changes in six different domains: sleep duration, social activity, mood, weight, appetite, and energy level. (In item 12 of the SPAQ, participants are asked about mood and behavior changes in these six domains related to seasons. These changes are rated for each domain on a five-step scale according to the amount of dependency on seasons. Value 0 means “no change,” while value 4 means “extreme change.”) High SPAQ scores indicate a more pronounced degree of seasonal changes.
2. Using SPAQ seasonality scores, a classification for seasonal disease states was performed according to subtyping, but only if item 13 showed worst well-being in winter months. (In item 13 of the SPAQ, participants are asked to relate certain patterns of behavior and well-being to the months with their maximal or minimal occurrence.) (Kasper et al., 1989) The SPAQ score 0–7 = no SAD; the SPAQ score 8–9 considered as “at least moderate problem,” and the SPAQ score 10 or higher considered as “at the most minor problem”: (subsyndromal) S-SAD; the SPAQ score 10 and higher considered as “at least moderate problem”: SAD.
3. Average sleep duration during a 24-h period in spring, summer, autumn, and winter was obtained from the SPAQ. (In item 16, participants are asked for the number of hours they sleep in a 24-h time span during each of the four seasons.)

### Degree of Pineal Calcification

**FIGURE 1:**
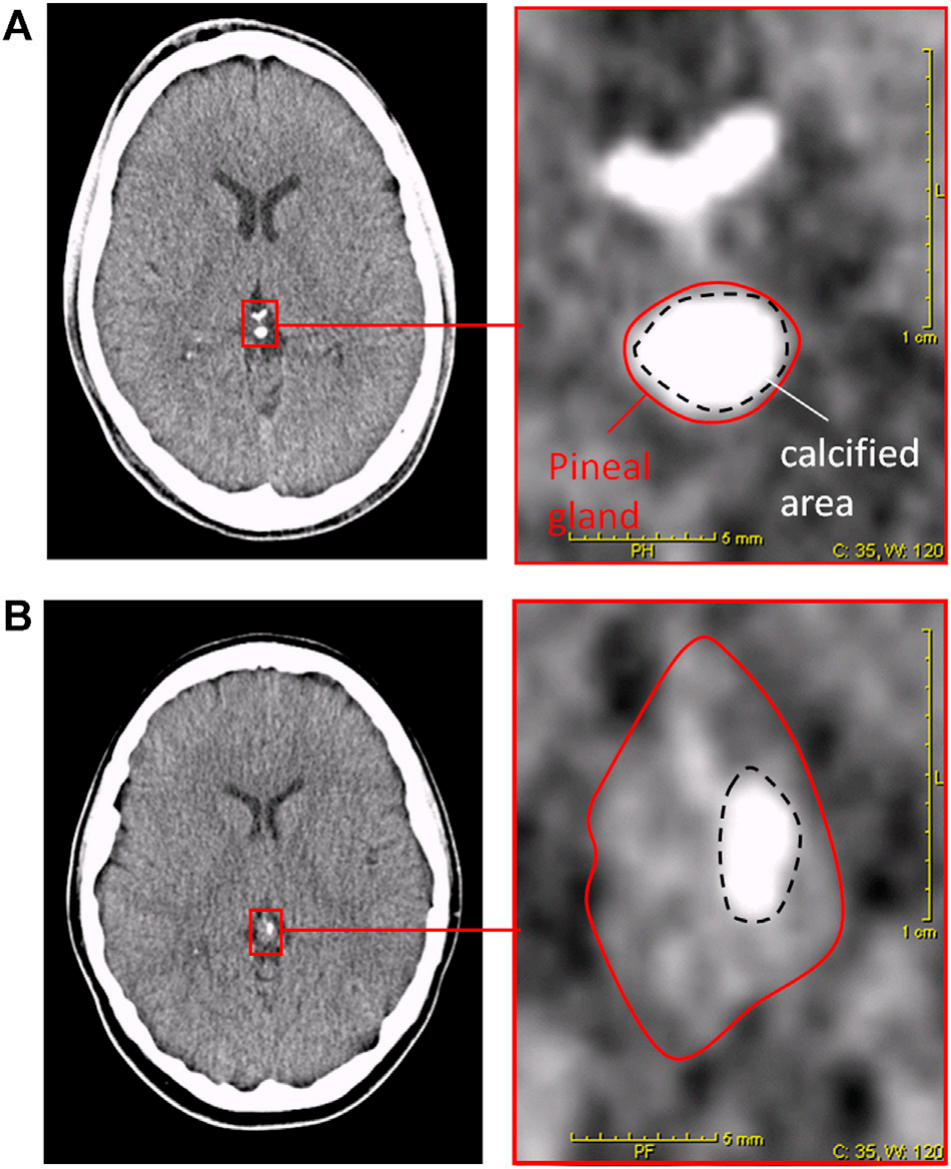
Degree of pineal calcification (DOC) in cCT-image analysis (Kunz et al., 1999)**.** Left pictures depict cCT 2-mm slices including the pineal gland. Right pictures depict magnification of red framed area in the left picture. Red solid lines surround the total pineal gland. Black dashed lines surround the calcified area of the pineal gland. Patient **(A)**: HE_max_ = 418; calcified portion: 80%; DOC = (3 + 3)/7 = 0.86. Patient **(B)**: HE_max_ = 164; calcified portion: 14%; DOC = (1 + 0)/7 = 0.14.

The DOC is a semiquantitative method that uses cCT imaging to determine the DOC as a marker for an individual melatonin deficit, as previously described (Kunz et al., 1999). In short, an automatic image analysis determines maximal density of pineal tissue in Hounsfield units. In addition, the proportion of uncalcified vs. calcified pineal tissue was visually determined. The two results were added to a score 0 = no calcification and 1 = completely calcified (Kunz et al., 1999). For examples of DOC determination on a cCT image, see Figures 1A,B. Every cCT was analyzed by two independent experienced scorers, who were blinded to clinical information and sociodemographic data about the subjects. For statistical analysis, consensus of the two raters was used.

### Statistical Analysis

Comparison of means for subjective sleep duration between seasons was performed using one-way ANOVA for repeated measures, and for single comparisons, t-tests for dependent samples including Bonferroni correction for multiple testing. To test the hypothesis of the association between DOC and the SPAQ seasonality score, first, Pearson correlation was performed, and second, partial correlation to control for age. Partial correlations calculate the amount of association between two variables (here, DOC and the SPAQ score), with the effect of a variable removed (here age), that is potentially moderating this association. If the extent of a numerical relationship between two variables is of interest, the Pearson correlation can give misleading results if there is another confounding variable that is numerically related to both variables of interest. This can be solved by controlling for the confounding variable, which is done by computing the partial correlation coefficient. In the third step, a regression analysis was performed to explore in greater detail the degree of potential age and interaction effects compared to the DOC effect on the SPAQ seasonality score. The 3D mesh plot presentation of the data was performed with the program SigmaPlot and smoothed with moving averages with sampling proportion of 0.1 and in the bandwidth method of nearest neighbors.

To further explore the expected ceiling effect on the interaction of DOC and seasonality with age, the partial correlation was repeated on cumulative subgroups of participants with growing age. *p*-values are generally based on two-tailed testing assuming a significance level of 0.05. Only some statistics were performed for the confirmation of known associations to show generalizability (associations of gender and age with seasonality) and then tested one-tailed. In these cases, two-tailed *p*-values are reported additionally.

## Results

A total of 97 healthy subjects (54 male; 43 female; range 18–68 yrs; mean age ± SD: 35.0 ± 13.1 yrs) were included. Distribution of age was not different between male and female participants (*p* = 0.667), although men were slightly younger on average (1.2 ± 2.7 yrs).

**FIGURE 2:**
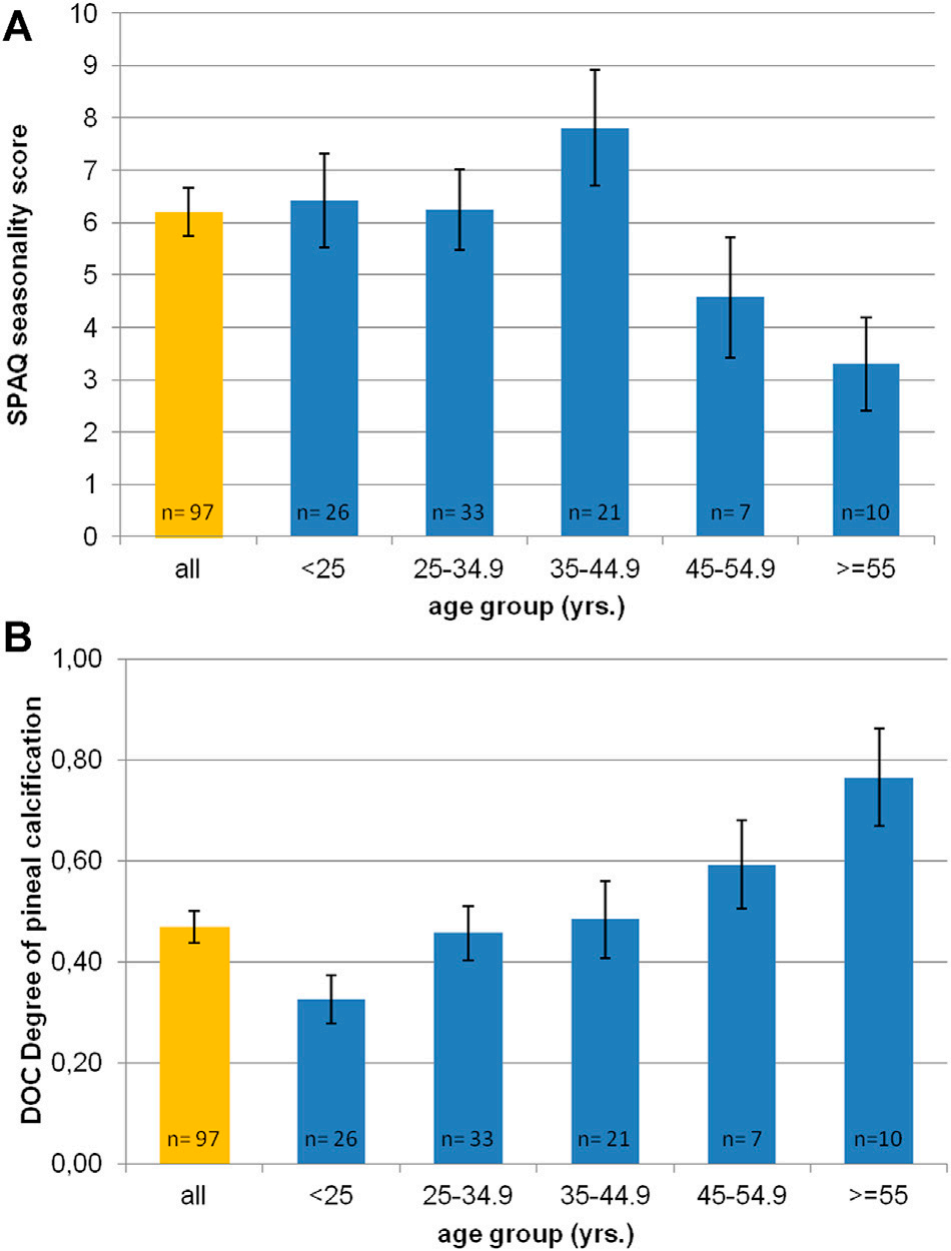
Age distribution of **(A)** SPAQ seasonality score and **(B)** DOC scores (degree of pineal calcification). Orange bar represents mean value of whole sample. Blue bars represent mean values per age-group (in 10 yrs spans). Error bars indicate standard error of mean.

Figure 2A shows the distribution of SPAQ seasonality scores with age. The mean SPAQ seasonality score was 6.21 (±4.50), whereas 17 participants (17.5%) experienced “no seasonality” (SPAQ score 1 and below), 9 participants (9.3%) fulfilled diagnostic criteria of suffering from SAD, and 17 participants (17.5%) fulfilled diagnostic criteria for subsyndromal SAD. To experience seasonality—as indexed by the SPAQ score—was more pronounced in women than in men (SPAQ seasonality score 7.8 ± 4.0 vs. 4.9 ± 4.5; *p* = 0.001) and significantly and negatively associated with age (r = −0.178; *p* = 0.04 one-tailed significance; *p* = 0.08 two-tailed significance).

The inter-rater reliability for determining DOC scores was excellent (r = 0.91; *p* < 0.01). Figure 2B shows the distribution of DOC scores with age. The mean DOC was 0.47 (±0.32). Female participants had significantly lower DOC than male participants (0.40 ± 0.32 vs. 0.52 ± 0.31; *p* = 0.03 one-tailed significance; *p* = 0.06 two-tailed significance). Age significantly correlated with DOC (r = 0.427, *p* < 0.0001). The older the participants were, the higher was the DOC.

**FIGURE 3:**
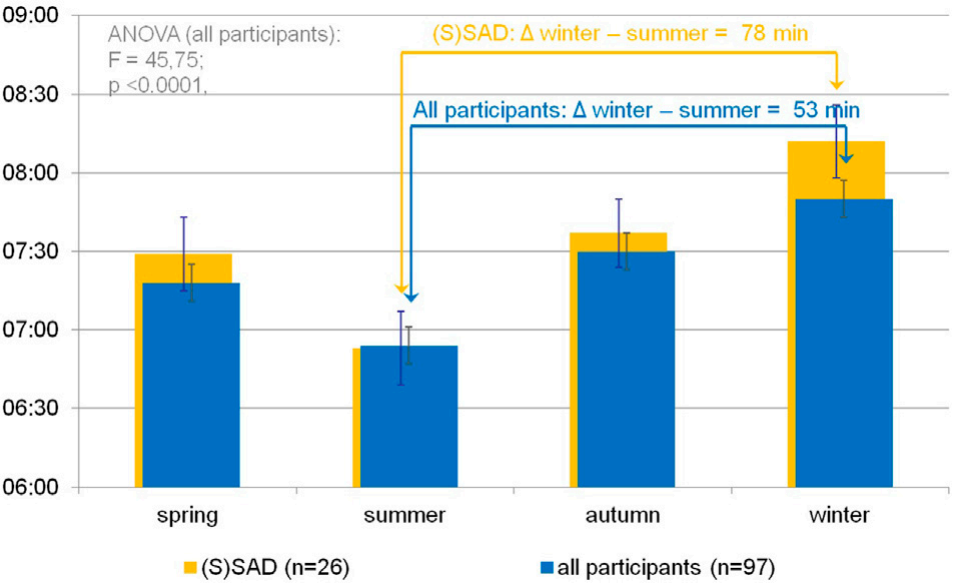
Seasonal variation of subjective sleep duration in an urban environment. Blue bars indicate self-reported sleep durations (hh:mm) per season as noted on the *x*-axis according to the Seasonal Pattern Analysis Questionnaire item 16 (Rosenthal and Wehr, 1984) in the whole sample. A one-way repeated-measures ANOVA shows significant differences in sleep durations between seasons. Single comparisons (*t* test) show significant differences between all pairs after Bonferroni correction for multiple testing (*p* < 0.01). Orange background bars show sleep durations in subgroup (participants categorized as SAD or subsyndromal SAD). Error bars indicate standard error of mean.

Figure 3 shows significant differences in the subjective sleep duration according to SPAQ between seasons (one-way repeated measures ANOVA: F = 45.75; *p* < 0.0001). Mean sleep durations (h:mm) per season were 7:18 (±1:05) for spring, 6:54 (±1:08) for summer, 7:30 (±1:04) for autumn, and 7:50 (±1.10) for winter. All single comparisons of sleep durations between seasons were significant after the Bonferroni correction for multiple testing (*p* < 0.05). Although the difference between summer and winter sleep duration was 53 min (±70 min) for the whole group, it was even more pronounced (78 ± 73 min) in the subgroup of participants classified as SAD or subsyndromal SAD.

**FIGURE 4:**
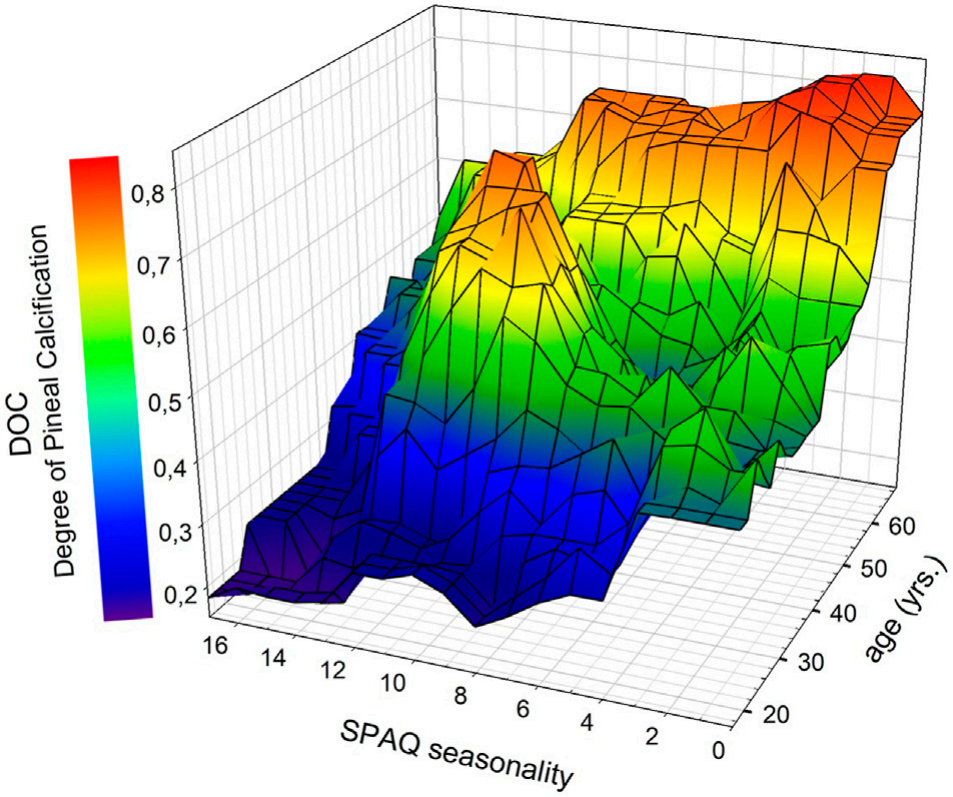
Associations between DOC scores (degree of pineal calcification), SPAQ seasonality scores, and age. Data were plotted with SigmaPlot as 3D mesh plots, smoothed with moving averages on sampling proportion of 0.1 using the bandwidth method of nearest neighbors. Colors represent DOC scores as indicated in the legend. * Significant age-independent correlation between seasonality and DOC (partial correlation: r_94_ = −0.214; *p* = 0.036). *Abbreviations:* SPAQ, Seasonal Pattern Assessment Questionnaire (Kasper et al., 1989): higher scores indicate more pronounced seasonality; DOC: higher scores indicate more pronounced calcification (Kunz et al., 1999).

Figure 4 illustrates the interaction of the SPAQ seasonality score (*x*-axis), DOC (y-axis), and age (z-axis) in a 3D plot. Visual inspection implies that in young adults (20 through 40 yrs; in the front part of the 3D plot), most DOC scores are in the lower range (0.0–0.4) and correlate with seasonality scores over the whole spectrum, whereas in older adults (45 and above; in the back part of the 3D plot), DOC scores are in the higher range (0.7 and above, colored in red and orange) and seasonality is limited to lower scores (right part of the 3D plot).

The DOC and SPAQ seasonality score were significantly correlated (r = 0.276; *p* = 0.008). After controlling for age, the correlation remained significant (partial correlation: r_94_ = −0.214; *p* = 0.036). Also, the regression model with the DOC and age as independent variables significantly predicted the SPAQ score (r^2^ = 0.076; F = 3.875; *p* = 0.025). The beta coefficient of the DOC was a significant predictor (Beta = −0.233; *p* = 0.036), whereas the beta coefficient of age was not (Beta = −0.079; *p* = 0.474). The beta coefficient is the slope coefficient of a certain variable in the regression line. When it is negative, the line is not growing but falling, with increasing values on the *x*-axis, which means there is an inversed association. So the SPAQ score is getting higher with smaller DOC values. After additional integration of the interaction term DOC*age, the model was not significant anymore. With increasing DOC scores, less seasonality was experienced by participants (see Figure 4).

**FIGURE 5:**
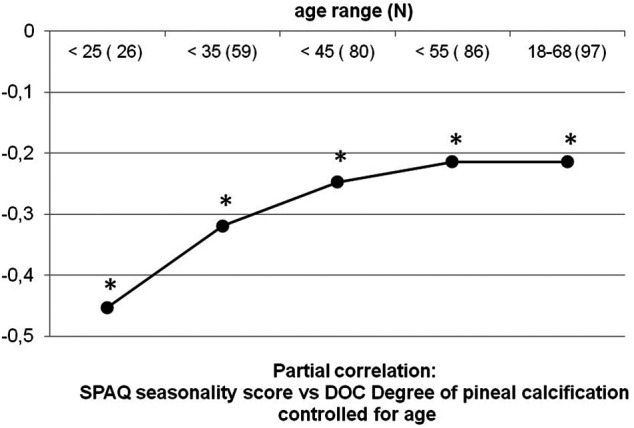
Partial correlations of DOC vs. seasonality in cumulative age-groups. Black dots represent partial correlations of the DOC vs. seasonality score (according to the Seasonal Pattern Analysis Questionnaire (Rosenthal and Wehr, 1984)) controlled for age. To explore a potential ceiling effect of age on the association of DOC and seasonality, partial correlations were repeated five times with cumulated samples stepwise including more participants with growing 10 yrs range age-groups. Sample sizes and age range are noted on *x*-axis. The correlation coefficient *r* is noted on y-axis. Correlations level out to smaller numbers with age, with a ceiling effect above 45 yrs old. * All correlations are significant (*p* < 0.05).

The exploration of a potential ceiling effect of age is displayed in table 1 and in Figure 5, which shows the development of the association between SPAQ seasonality and DOC controlled for age, starting with a sample of young participants and subsequently adding participants of growing age. In young participants of less than 25 years, the association of SPAQ seasonality and DOC controlled for age is pronounced with higher DOC scores, indicating less seasonality (partial correlation: r_23_ = −0.453; *p* = 0.023; *n* = 26). Subscript numbers after r_xx_ display degrees of freedom. Degrees of freedom grow because the denoted age-groups are growing in sample sizes when the next age-group is added. Adding groups of participants with increasing age (10 yrs) results in a leveling out to a smaller degree of association: (group < 35 yrs: r_56_ = −0.319, *p* = 0.015, *n* = 59; group < 45 yrs: r_77_ = −0.247, *p* = 0.023, *n* = 80; group < 55 yrs: r_83_ = −0.214, *p* = 0.048, *n* = 86; group all ages: r_94_ = −0.214, *p* = 0.036, *n* = 97). When analyzing participants in the age-group > 45 yrs alone, no statistical association was found (r_14_ = −0.081; *p* = 0.767; *n* = 17).

**TABLE 1:** Partial correlations of DOC vs. sesonality in cumulative age groups.

Age-group (yrs.)	N	Degrees of freedom	r (partial correlation)	P
<25	26	23	−0.453	0.023
<35	59	56	−0.319	0.015
<45	80	77	−0.247	0.023
<55	86	83	−0.214	0.048
All	97	94	−0.214	0.036
>45	17	14	−0.081	0.767

## Discussion

The data presented here confirm various earlier reports: women experience seasonality of behavior more often than men; seasonality is experienced more often by younger than older adults; about 25 percent of the adult population suffers from seasonality to an at least moderate extent (SAD or sub-SAD) (Kasper et al., 1989; Wehr et al., 1995; Magnusson and Boivin, 2003); and DOC scores increase with age (Kunz et al., 1999). Thus, our cohort seems to be generalizable to the regular population. This study confirms earlier preliminary data (Kunz et al., 2001) that seasonality of behavior in humans depends on the functioning of the pineal gland: 1) an advanced DOC as indicator of hypopinealism is associated with low seasonality of behavior in humans; and 2) the magnitude of this seasonality is that a regular adult population, living in an urban environment at Berlin latitude, report a variation in sleep length of about 1 h over the year. As such, data of this study may supply the missing link between low melatonin and low seasonality (Wehr, 1997) by the introduction of high DOC scores to the picture.

The size and the weight of the human pineal gland vary between individuals by about 20-fold, which is paralleled by a 20-fold variation in melatonin excretion. The calcification process of the human pineal gland seems to start with birth and is visible in CT scans in around 80 percent of young adults, with growing extent thereafter (for review Tan et al., 2018). A reversal of calcification of the pineal gland was never reported yet. We have shown in various populations that a high DOC score is associated with, for example, Alzheimer’s disease (Mahlberg et al., 2008) and circadian modulated REM sleep parameters in polysomnography performed in patients with insomnia (Mahlberg et al., 2009). The total amount of melatonin excretion is neither associated with clinical parameters—except in Alzheimer’s disease—[for review Mahlberg et al. (2008)] nor with the size of uncalcified pineal volume (Mahlberg et al., 2009). We had shown that in humans, not the size of the pineal gland but the uncalcified pineal volume alone correlates with melatonin excretion rates (Kunz et al., 1999; Mahlberg et al., 2009). Thus, the individual pineal deficit marker DOC indicates individual pineal functionality with high DOC scores representing hypopinealism and medium or low DOC scores representing eupinealism.

The mechanism of pineal calcification is not fully understood. Animals have been reported to have a calcified pineal gland as well (Welsh, 1985). On the other hand, the magnitude of this calcification seems to be unique in humans, and most animals reported to develop a pineal calcification are living in human neighborhood [for review, see (Tan et al., 2018)]. Thus, one hypothesis would be that human lifestyle contributes to the calcification process. First rank candidates would be shift work and the application of unnatural lighting regimes including spectrum, intensity, and timing over the day and year.

Seasonal variation in behavior is known to occur in young adults more often than in older subjects (Wehr et al., 1993; Magnusson and Boivin, 2003). It is also well known that melatonin excretion rates decrease with age (Mahlberg et al., 2006), which is paralleled by a reduced size of uncalcified pineal volume in the individual (Kunz et al., 1999; Mahlberg et al., 2009). Data presented in this study confirm earlier preliminary results, showing that phenomena of seasonality and DOC are interrelated (Kunz et al., 2001). In line with the existing literature, seasonality scores level out to very low scores above the age of 40–50 years (Kasper et al., 1989) and DOC levels out to high scores (Kunz et al., 1999; Mahlberg et al., 2008). This ceiling effect results in no statistical significance between DOC and seasonality scores when this group of advanced age is studied alone. In sharp contrast and again in line with the literature, younger adults do experience seasonal variation of behavior, paralleled by a progressive decrease in melatonin excretion, which is caused by increasing DOC.

The question arises whether seasonality or non-seasonality indicates physiological or pathological phenomena, respectively. A need for human hibernation in most areas of the world does not exist. Hibernation in animals is not a state of, for example, restoration, but rather a mode of energy saving (Pittendrigh, 1993). Accordingly, reports indicated in humans that, for example, serotonin turnover by the brain is reduced to 20–30 percent in winter as compared to summer (Lambert et al., 2002). Thus, seasonality of behavior in humans was suggested to represent human hibernation (Whybrow and Bahr, 1988; Wehr et al., 1993). In contrast, why does the crest of suffering from seasonal phenomena occur at the end of February, instead of around the shortest day, which is in December? One explanation could be a *living on reserve* in the beginning until a *running on empty* at the end of winter period.

It is surprising and impressive that our population, restricted to the constraints of working hours and living in an urban environment, experience a variation in the sleep length of about 1 hour over the year. Possibly, this represents only the tip of the iceberg. In modern societies, people are forced to keep the same time schedules in winter as in summer because of scheduled school times and work hours. It would be interesting to investigate summer–winter changes in, for example, the sleep length and quality without the constraint of morning awakening by the alarm clock. Another factor is the type of light (predominantly artificial vs. predominantly natural) for people in the same latitude. Possibly, people living in rural areas may even experience larger seasonal variations than those living in an urban environment.

Not all people with high seasonality scores subjectively suffer as indicated by the large group of sub-SAD (definition includes “SAD score above 9, but not suffering to an at least moderate extent”). We hypothesize that the extent of affection by symptoms of seasonality during the months of winter depends on the actual amount of sleep obtained in comparison to the amount of sleep needed. People with eupinealism, indicated by low DOC scores, experience an increased sleep need during winter. Only when this additional sleep need is unmet, patients will suffer from insufficient sleep syndrome. On the other hand, the difference in the sleep duration between winter and summer would be even longer than observed in our study. Data of our study suggest the necessity to meet seasonal variation of school and work start.

### Limitations

Telephone interviews were performed up to 3.5 years after the cCT investigation. Even though participants were asked to refer their seasonal experience to the time prior to emergency contact, impaired memory could be a factor in a part of the group. On the other hand, people usually recall in a SPAQ interview longer periods than the year past when asked for their seasonal variation in behavior. We included elderly patients even though a ceiling effect for increasing DOC scores as well as reduced seasonality experiences are both well-known factors with age. The effects reported here were more pronounced when age was not controlled for.

Our population was not checked for the lighting condition they lived in. The melatonin excretion period length—signaling “night” to the organism—varies significantly under laboratory conditions, with light–dark schedules mimicking natural summer vs. winter conditions. In contrast, the melatonin excretion period length does not differ between summer and winter under natural urban light conditions, which is due to the urban situations including more artificial than natural sunlight (Wehr et al., 1995).

In conclusion, data presented here elegantly explain the lack of experiencing seasonal variation of behavior in humans by the increasing DOC. Open questions for future research include: do people experiencing seasonal variation of behavior cope better with seasonal changes in lighting than those without seasonality resulting in better health in the long run? Why do only some young athletes perform differentially over the year with performing worst during late winter? What are the mechanisms triggering the process of hypopinealism as indicated by high DOC scores?

## Data Availability

The original contributions presented in the study are included in the article/Supplementary Material; further inquiries can be directed to the corresponding author.
